# Enemata

**Published:** 1871-08

**Authors:** Robert Battey

**Affiliations:** Rome, Ga.


### ENEMATA.

BY ROBERT BATTEY, M. D., ROME, GA.

The enema or clyster is a form of medication which has been handed down to us from remote antiquity, and still holds an important position amongst the resources of our art, attested by the experience of a long line of disciples of Æsculapius, Hippocrates, and Galen, even down to our own day; in general use and esteem among all classes, and in all nations of the earth, where medical science, be it ever so rude, is at all known ; the efficacy and value of rectal medication need not be insisted upon.

Among the Egyptians, at a time when physic appears to have been little else than a collection of absurd superstitions, the clyster was known and valued, and is said to have been suggested to them by the sacred Ibis, a bird, whose habit it was to give itself clysters with its bill. Hippocrates, says a writer of the past century, used suppositories and clysters. "The former were compounded of honey, the juice of the herb mercury,* of nitre, powder of colocynth, and other sharp ingredients, to irritate the anus. These they formed into a ball or into a long cylindrical mass, like a finger. The clysters he made use of for sick people, were sometimes the same with those already mentioned as preventatives for people in health. At other times he mixed the decoction of herbs with nitre, honey, and oil, or other ingredients, according as he imagined he could, by that means, attract, wash, irritate or soften. The quantity of liquor he ordered was about thirty-six ounces ; from which it was probable he did not intend that it should all be used at one time."

Dr. John Quincy, in his *Lexicon Physico Medicum*—London, 1794, says, "the words enema, clyster, and lotion, are equivalent to each other, and signify any liquid medicine injected into the anus."

The Edenburgh Encyclopedia, of about the same date, defines

*Chenopodim Bonus Henricus.

"clyster as a liquid remedy, to be injected chiefly at the anus, into the larger intestines. It is usually administered by the bladder of a hog, sheep, or ox, perforated at each end, and having at one of the apertures an ivory pipe, fastened with pack-thread. But the French and sometimes the Dutch, use a pewter syringe, by which the liquor may be drawn in with more ease and expedition than in the bladder, and likewise more forcibly expelled into the large intestines." "The English introduced a new kind of clyster, made of the smoke of tobacco, which has been used by several other nations, and appears to be of considerable efficacy when other clysters prove ineffectual, and particularly in the illiac passion,* in the hernia incarcerata; and for the recovery of drowned persons."

The ivory pipe above alluded to has been handed down to the present generation. The writer well remembers in his first apprenticeship in a drug-store, those small pipes of ivory or bone, roughened at one end, that they might be tied into a bladder, as a common article of sale under the name of "fistua eburis." The old pewter syringe in use among the French and the Dutch at the close of the last century, is still much employed, and for the administration of small enemata on account of its simplicity, cheapness, and convenience, seems destined to be held in equal esteem in the future. For general purposes, however, many improvements have been successfully introduced, until we now have the hard rubber syringe, modeled upon the old French pattern, and the various styles of self-rubber instruments, with elastic bulbs, which appear to be as perfect in their adaptation as can be desired.

No one who has not been accustomed, in important cases, to superintend in person the administration of enemata, can well realize the beneficent potency of the remedy in many a fearful crisis with the sick. Ignoble as some esteem the service, there is always room for the display of tact and skill, and often demand the greatest coolness and judgment to rescue life in emminently impending danger. The carelessness with which an enema is too often ordered at the hands of an ignorant nurse; the indifference manifested as to its composition, its temperature, its bulk, and its manner of exhibition, evidences not only want of care for the comfort and health of the patient, but positive ignorance of the power invoked in its capabilities for good or harm. It is questionable whether a student of medicine ought to receive his diploma until he has demonstrated clinically his capacity to prepare and administer the ordinary enema *secundum artem.*

In the administration of enemata in general, a few rules are to

*Intussusception.

be observed, and first, the hands should be well washed with soap, to soften them and insure their entire cleanliness in this, as in all manipulations about the openings of the body. The preparation of the enema may ordinarily be made in a bowl or pan, and the syringe (a modern one) is to be operated for a few moments, throwing the fluid back into the bowl, until *all air* is expelled from the instrument; leaving the rectal tube beneath the surface to prevent ingress of air.

For convenience of administration the patient may be placed upon the left side with the back near the edge of the bed, the knees drawn up, and the body and limbs covered with the usual bedclothing. No one can consider himself an adept in the art, who is not prepared at all times, to administer an enema successfully in *any position* the patient may assume. To *uncover* a patient for this service is incompatible with the self-respect of the physician, patient and nurse. It is an inexcusable barbarism, which is chargeable with much of the disgust so commonly felt for this valuable form of medication.

These preliminaries having been arranged, the administrator seats himself at the bedside with the bowl conveniently at hand; anoints the forefinger of the right hand with oil or lard, and placing the tip of the finger in the sulcus of the nates adjacent to the coccyx, draws it forward upon the perinem until the anus is felt, when the entire first joint of the finger is gently passed into that opening. Taking now the recal tube of the syringe in the left hand, and directing its point strongly upon the engaged finger, pass the tube slowly into the rectum, withdrawing at the same time, the finger as the tube passes in. This manipulation, with practice, is executed rapidly and certainly, and without pain or even discomfort to the subject.

It should always be observed, and especially in the male, that the rectal tube, after passing the sphincter muscle, is directed backward and upward in the axis of the canal, that the point may not press painfully upon the prostate gland or uterus, as the case may be. If the old fashioned pewter syringe is used, when, in proper position, its direction will be upward and backard, toward the sacrum, not in a line with the axis of the body; much less upward and forward toward the bladder, as one may too often see.

If a small syringe is to be used, the manipulation is the same, and the use of the finger as a guide is even more important, since the passage of a small pipe into the rectum is both difficult and painful without such guide, whilst no pain is given when the point of the pipe presses only upon the finger of the operator.

For the present purpose enemata may be conveniently classified as follows: 1. purgative; 2. emolient; 3. anodyne; 4. refiigerant; 5. styptic; 6. distensile; 7. exciting; 8. relaxing; 9. nutritive.

*Purgative* enemata are designed merely to unload the lower portion of the alimentary canal. For an adult, they vary in bulk from eight to thirty-two ounces; they are composed of tepid water rendered somewhat stimulating by the addition of soap or salt, sometimes infusion of senna, or colocynth, also sulphate magnesia, castor oil, spirits turpentine, or watery solution of aloes. It is questionable whether any desirable advantage is derived from such combinations, in any but very exceptionable cases; warm water and the common turpentine bar soap may be so varied in bulk and temperature of fluid, and in quantity of the soap, as to meet the exigencies of any case of simple constipation. By increasing the bulk of fluid it penetrates higher up the canal, distending the parieties, lubricating and loosening the accumulations, and exciting to active contraction the muscular coat of the intestines; by elevating temperature, and augmenting the quantity of soap, likewise, muscular contraction is excited to any desirable degree.

When castor oil or turpentine is used, it should be first soaked into a rag and washed out with strong soap into the enema water, to effect an intimate and perfect dissemination of the oil.

*Emolient* enemata may be employed to allay irritation in the canal itself, or in the bladder, uterus, or other contiguous organs or structures. They are compounded of simple luke-warm water, little, if at all, above the temperature of the body, or of mucilaginous solutions of starch, gum or other demulcent. In bulk they may vary from two to eight, or even twelve ounces, and should be thrown into the rectum very slowly, and with special precautions to exclude all air from the syringe.

*Anodyne* enemata are much employed for the controll of irritation and pain in any of the abdominal organs, to check diarrhœa and dysentery, to restrain nausea and vomiting, to stop impending abortion, as well as to tranquilize the general nervous system. They are composed of the same bland fluids used as emolients,—with the addition of some one of the preparations of opium, or of the narcotic extracts, and more recently hydrate of chloral, which may be eligibly exhibited in this manner.

The bulk of anodyne enemata should always be small; in general, from one to two ounces; and used in a syringe of the old patern, hard rubber, metal or glass; suited in size to the bulk to be given. It is especially important that air be excluded, and to secure this, the syringe should be opened, a finger placed upon the orifice of the pipe, a teaspoonful of the demulcent fluid, put into the syringe, the anodyne ingredient added next, and the syringe filled with the demulcent as near the top as will barely leave room for the lower end of the piston, which should now be put in place and forced down until a single drop of the fluid escapes past its packing, to indicate that air is well excluded, when the cap is screwed down and the instrument is ready for use. It is a convenience to have the piston mounted with a ring to receive the forefinger, while the barrel is held between the thumb and the lesser finger of the left hand; by which the injection may be made with the one hand.

If this enema is expelled, it should be soon repeated, and is more likely to be retained than at first. It ought to be patiently persevered with, and if repeatedly returned, a searching inquiry should be made into all the steps of the manipulation, to ascertain if the fault be not with the manipulation, as it usually is in such case.

*Refrigerant* enema are usefully employed in dysentery, enteritis, gastritis, hemorrhoids, and in intestinal, vesical and uterine hemorrhages. They consist of simple cold water, or ice water, and are to be introduced very slowly into the rectum to ensure retention. If the enema be expelled, the second or third will likely be retained.

*Styptic* enemata are use to controll intestinal hemorrhages, and have power also, by absorption, to restrain hemorrhages generally, when stypics by the mouth are inadmissable. They are composed of cold water, with one of the per-salts of iron, alum, acetate lead, or the various vegetable astringents ; especially gallic acid, which is probably the safest and most prompt and effective of all. The bulk of the enema is governed by the seat of the hemorrhage ; being small if this be low down in the canal or in some other organ, and larger in proportion as its seat is higher up.

*Distensile* enemata, when judiciously and, at the same time, *boldly* employed for the removal of intestinal obstructions, can scarcely be over-estimated. The great power of the remedy, coupled with its comparative harmlessness—is not so generally known and utilized as it ought to be. For this purpose the enema must be decidedly unstimulating ; it should be even emolient in its propties ; warm water at about the temperature of the blood is appropriate. The bulk required is ordinarily larger, and an abundant supply, at least four gallons ought to be at hand, that there be no lack. The syringe must be arranged for continuous action, as the modern styles are, and must also be strong and durable ; a capacious vessel, likewise, must be in readiness to receive the evacuation ; and the bed suitably protected against accident, that it be not soiled.

The enema is to be administered in the usual manner, the patient being in the obstetrical position, upon the left side. The important issue hanging upon the result fully warrants the placing of the patient so as to be easily accessable to the operator, and even, if he be inexperienced, the exposure of the nates ; though an expert will not find the latter expedient either necessary or desirable.

When complaint is made of distension the injection is to be suspended a little, until the feeling of tenesmus passes off, and then resumed slowly and gently, while the patient is urged to retain it as long as possible. When the power of retention is over-taxed and seems about to give way, it ought to be assisted by a folded napkin firmly pressed over the anus, around the tube by the left hand, or better still, by both hands of an assistant. When the combined powers of the patient and assistant will no longer prevent the escape of the fluid, the syringe should be operated rapidly and forcibly for a few moments, until the distress of the patient peremtorily demands a cessation; then, and not till then, is he allowed to get up.

In the event the obstruction is not removed at the first essay, the patient is allowed a few hours rest, render the tranquilizing influence of opium or chloral, and the effort then renewed, while he is yet under its influence. Chloroform, by inhalation, is sometimes required to assist the toleration of the method.

It is scarcely necessary to add the precaution that the full power of the distensile enema is never to be invoked *late* in a case of obstruction, or when there is reason to apprehend the walls of the tube have been materially weakened by resulting inflammation.

*Exciting* enema, have for their object a general arousing of the vital powers in coma, and sometimes in collapse. They offer a ready means of strongly impressing the system with local, as well as diffusible stimulants. They give increased strength to the action of the heart, against nerve force, and elevate sinking temperature.

They are composed of *hot* water impregnated with such stimulant as may be at hand in the emergency, and which seems best suited to the particular case. Often promptness in the application of the remedy is of vastly more importance than the choice of stimulant. Among the articles which may be mentioned in this connection, are mustard, cayenne, brandy and spirits turpentine. The quantity of these ingredients, as well as the temperature of the liquid, may be increased greatly in extreme cases of coma, not only with impunity, but with the happiest results. No rule can be laid down for the quantity of such injections, but in general they should be so large that the fluid may reach high up the canal, and be the more promptly returned, to be repeated again and again, as may be required.

*Relaxing* enemeta are designed to overcome intestinal spasm, strangulated hernia, and spasmodic strictures. They are composed of tepid water, of soothing temperature, with assafœtida emulsion, tincture lobelia, decoction tobacco, &c.,—or they may consist simply of tobacco smoke. The latter may be collected by burning tobacco in a stone jar properly covered, from which it is to be drawn into the syringe and forced into the rectum. A safer and more eligible form of tobacco enema is weighed quantities of Scotch snuff, dipped in the warm water. This remedy should always be used with caution, and is to be given in fractional quantities—time being allowed to mark the effect of each successive portion. Should the general depression pass beyond a safe limit, an exciting enema ought to follow, with the two-fold view of removing the relaxant and rallying the patient.

Of *nutritive* enemata little need be said; their value, in suitable cases, is well known, and in general use. They consist of nutritious liquids, which, when to be long used, ought to represent not only meats, but vegetable juices likewise, with sugar, salt in small proportion, and wine or brandy, to promote absorption. Such enemata must be free of air, and slowly introduced to secure their long retention. Laudanum or morphine may be required when the intestine is irritable.

